# Probiotic supplementation improves well-being and anxiety in healthy women: An exploratory, randomized, double-blind, placebo-controlled study

**DOI:** 10.1080/29933935.2025.2543125

**Published:** 2025-09-05

**Authors:** Daryn R. Michael, Niall Coates, Joshua Kerry-Smith, Daniel A. John, Eleri. Hulme, Lauren Owen, Sue F. Plummer

**Affiliations:** a Research & Development, Cultech Limited, Port Talbot, UK; b Human Behavioural Neuroscience Laboratory, Department of Psychology, School of Humanities and Social Sciences, Leeds Beckett University, Leeds, UK

**Keywords:** Probiotic, women, well-being, anxiety, microbiota

## Abstract

The gut microbiota plays an important role in the maintenance of health and well-being throughout the life course but little is known about the impact of these changes on the balance of the microbiota and the well-being of women during their midlife transitional phase. In this exploratory, randomized, double-blind, placebo-controlled study, women (45 to 65 years old) received either a daily dose of probiotics or placebo for 4-months. Measurements of overall well-being included anxiety, depression, quality of life and sleep quality, gastrointestinal discomfort and changes to the fecal microbiota composition(ClinicalTrials.gov: NCT06507111). The women in the probiotic group showed a 40% reduction in anxiety, an 18.5% improvement in quality of life and indications of improved sleep, resulting in a significant 17.5% improvement in overall well-being compared to those in the placebo. Incidence rates of indigestion and stomach pains were reduced and *Blautia* sp. KLE 1732 was less abundant in the probotic group at the end of the study. Daily probiotic supplementation may support the well-being of women in their 40s − 60s.

## Introduction

The gut microbiota is a vast community of microorganisms that forms part of a complex communication network linking the gastrointestinal tract with the immune system and central nervous system.^1^ The gut microbiota plays an essential role in maintaining human health and supporting many physiological processes via metabolic and immune modulation, barrier maintenance and colonization resistance.^1^ The composition of the gut microbiota can be disrupted by numerous factors such as diet and lifestyle, the environment and antibiotic usage^2^ but there is also a natural shift over the course of life with the largest changes occurring early in life reaching stability around the age of 4 and with a pronounced sexual dimorphism developing from puberty.^3^

Women experience hormonal changes as they age and enter the menopause transition (MT) that can start many years prior to menopause.^3^ During the MT period (generally after 40 years old), over 80% of women suffer hormone-related changes including vasomotor symptoms (hot flushes and night sweats), gastrointestinal and genitourinary symptoms, mood and cognitive changes and sleep disturbances.^4^ There is a direct relationship between the levels of sex hormones (estrogen and progesterone) and the composition of the gut microbiota and the age related changes in women and this manifests as decreased gut microbial diversity.^3^ It has been suggested that a higher gut microbiota diversity could promote hormone retention and optimization of the levels of systemic sex hormones.^3^ It is possible that modulation of the gut microbiota in women during this transitional disruptive period of life could provide a means of alleviating some of the adverse symptomology that is experienced.

Modulation of the composition of the gut microbiota has been achieved in some instances with probiotics (defined as “live microorganisms that, when administered in adequate amounts, confer a health benefit on the host”^5^), however, the number of intervention studies specifically targeting mid-life aged women is limited. Probiotic supplementation has been linked with increased circulating estrogen levels,^6^ improved vascular function^7^ and relief from a range of age-related symptoms such as anxiety, depression, hot flushes and fatigue.^8–10^ The Lab4P probiotic comprising strains of lactobacillus and bifidobacterium has shown the ability to improve gastrointestinal status in both healthy adults and in a cohort of female irritable bowel syndrome sufferers with mood and quality of life markers also improved in the IBS cohort.^11–13^

In this study, we took healthy women aged between 45 and 65 and assessed the impact of 4 months of daily supplementation with the Lab4P probiotic. A range of outcomes were recorded including anxiety and depression, quality of life, sleep quality, incidence rates of gastrointestinal discomfort and changes in the composition of the gut microbiota. The aims of this exploratory study were to *i*) assess the potential application of this probiotic for the improvement of overall well-being in the women and *ii*) provide a basis for future menopause targeted work.

## Methods

### Study design

An exploratory, double-blind, randomized, single-center, placebo-controlled superiority study with the equal allocation of 50 participants between two parallel groups.

### Study approval

The study was performed in accordance with the principles of the Declaration of Helsinki by Comac Medical (Sofia, Bulgaria). Ethical approval was granted by the Ethical Committee of Comac Medical (Reference: #1/02.07.2024). The study was prospectively registered with ClinicalTrials.gov on 03/07/2024: NCT06507111.

### Recruitment & randomization

All participants entering the study provided written informed consent and were sequentially assigned to one of the 2 groups in a 1:1 ratio (block-size of two). The randomization code was generated using online software (www.sealedenvelope.com) and the study intervention was randomized at the point of manufacture, prior to its arrival at the trial site. The allocation sequence was not available to any member of the research team. Participants received financial compensation for taking part.

The inclusion criteria were: women aged 45 to 65, willing to provide fecal samples, body mass index (BMI) < 30 kg/m^2^ and willing to maintain a normal diet and lifestyle throughout the study. The exclusion criteria were: probiotic intake during the 30-days prior to the study, oral antibiotic use during the 90-days prior to the study, premature menopause (onset before the age of 40), receiving hormone replacement therapy, routine shift work, pregnant or planning pregnancy or given birth in the last 3 months, unexplained loss of weight over recent months, immunodeficient/immunosuppressive therapy, and diagnosed diabetes/cardiovascular disease/cancer/dementia.

### Study product

The probiotic product (Lab4P) comprised white capsules containing *Lactobacillus acidophilus* CUL60 (NCIMB (National Collection of Industrial, Food and Marine Bacteria) 30157), *Lactobacillus acidophilus* CUL21 (NCIMB 30156), *Lactiplantibacillus plantarum* CUL66 (NCIMB 30280), *Bifidobacterium bifidum* CUL20 (NCIMB 30153) and *B. animalis* subsp. *lactis* CUL34 (NCIMB 30172), 50 billion colony-forming units (cfu)/day, Vitamin C (80 mg/day), Vitamin D (400 IU/day) and Zinc (10 mg/day) on microcrystalline cellulose; the matched placebo capsules contained microcrystalline cellulose only (Cultech Ltd, Port Talbot, UK). Products packaged into induction-sealed high-density polyethylene pots (60 capsules per pot). The participants took one capsule (with or following food but not with hot drinks) daily for 4 months, stored capsules in a refrigerator and returned unused capsules at the end of the study for compliance monitoring.

### Study aims

The aims of this exploratory well-being study were to assess changes in anxiety, depression, quality of life and sleep quality, the occurrence of gastrointestinal and other common discomforts and the fecal microbial composition.

### Data & sample collection

**Figure 1.**
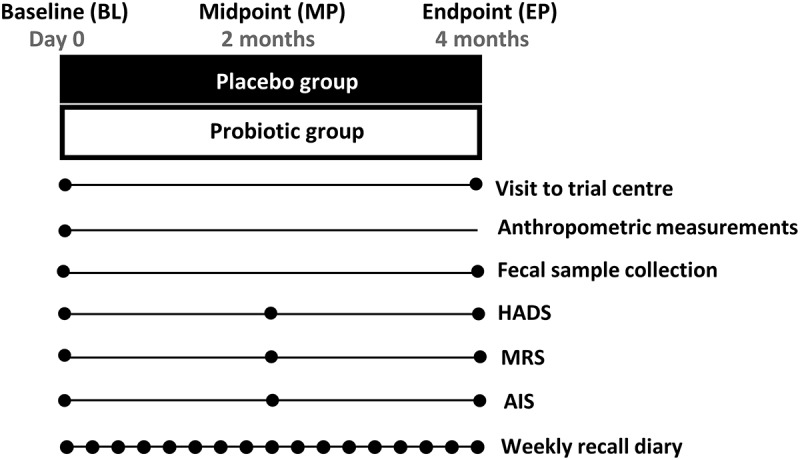
Sequence of data and sample collection. Abbreviations: HADS, Hospital Anxiety and Depression Scale; MRS, Menopause Rating Scale; AIS, Athens Insomnia Scale.

The sequence of data and sample collection during the study is shown in Figure 1. Anthropometric measurements were taken at baseline. Fecal samples were collected within 48 hours of the baseline and endpoint (4 months). Well-being questionnaires were completed by participants at baseline, midpoint (2 months) and endpoint. Recall diaries were completed weekly.

#### Anthropometric measurements

Body weight was measured using a calibrated column scale (Seca 709, Hamburg, Germany) after the removal of shoes and jackets. Shoes were removed before height measurement. Blood pressure was measured after five minutes’ rest (seated) using a calibrated blood pressure monitor (Omron, Kyoto, Japan).

#### Well-being questionnaires

##### The Hospital Anxiety and Depression Score (HADS)

HADS is a 14-item questionnaire (7 items for Anxiety and 7 items for Depression) scored on a 4-point Likert scale with 0 as the least problematic and 3 as the highest level of problem, giving a maximum score of 42 with 21 for each of Anxiety or Depression.^14^

##### The Menopause Rating Scale (MRS)

MRS is an 11-item questionnaire scored on a 5-point Likert scale (0 = no problem to 4 = severe problem), giving a maximum score of 44.^15^

##### Athens Insomnia Scale (AIS)

AIS is an 8-item questionnaire scored on a 4-point Likert scale with 0 representing good sleep quality and 3 representing the poorest sleep quality, giving a maximum score of 24.^16^

For use in the study, MRS and AIS were translated from English-to-Bulgarian language by professional translators (validated translations were not available).

##### Overall Well-Being Score (OWBS)

Scores from the HADS Anxiety, HADS Depression, MRS and AIS questionnaires were combined into an OWBS. For each participant at each time-point, questionnaire scores were standardized to a 0–100 scale with the formula:

$$\mathrm{Standardised}\;\mathrm{score}=\left(\frac{X-X\min}{X\max-X\min}\right)\;x\;100$$

where *X* is the participant’s score, and *X*min and *X*max represent the minimum and maximum values, respectively, observed for the entire dataset.

The values for the standardized questionnaire scores were averaged to generate an OWBS per participant. An OWBS value of 0 for a participant indicates the best well-being status, while a score of 100 represents the lowest well-being status.

### Digestive and other discomforts

Measured retrospectively using a *weekly recall diary* (Supplementary Figure S1) recording the number of days out of the past 7 experiencing symptoms of constipation, diarrhea, bloating, stomach pain, indigestion, sore throat, headache, muscle ache and colds.

### Menstruation and antibiotic and pessary use

Measured retrospectively using the *weekly recall diary* recording the number of days out of the past 7 with menstruation, oral antibiotics use and/or non-hormonal vaginal pessary/cream use.

### Statistical analysis

Between group differences in scores for HADS Anxiety, HADS Depression, MRS, AIS and OWBS were analyzed using mixed-effects models (*glmmTMB* R package (v1.1.10)).^17^ Fixed effects were group, time-point and an interaction between group and time-point. Age, BMI, menstruation and baseline questionnaire score were included as covariates, and the subject as a random effect. Model residuals were assessed for homoscedasticity and normality using the SimulateResiduals function of the *DHARMa* R package (v0.4.7).^18^ Data is presented as adjusted means (Estimated Marginal Means/EMMs) with 95% confidence intervals that were obtained using the *emmeans* R package v1.10.7.^19^

Cumulative Link Mixed Models (CLMMs) for ordinal outcome data, implemented using the *ordinal* R package v2023.12–4.1,^20^ were used to assess the item-level (individual question) responses from questionnaires. Fixed effects included group, time-point, and their interaction. Covariates were age, BMI, self-reported menstruation, and the baseline score for each item/question and the subject was included as a random effect. Results are reported as adjusted Odds Ratios (ORs) with 95% confidence intervals (CIs) representing the odds of reporting a lower score (i.e. improvement) in the probiotic group divided by the odds in the placebo group. An OR > 1 indicates that participants in the probiotic group were more likely to report a lower score, whereas an OR < 1 indicates they were less likely.

Incidence Rates (IR) of digestive and other discomforts for each participant are expressed as the number of days with a symptom divided by the number of months in the intervention period. Between group differences in IR were analyzed using linear models, with group as a fixed effect and age, BMI, menstruation and pre-intervention incident rate (based on the prior week at the first day of the trial) as covariates. The adjusted mean IR with 95% confidence intervals was obtained using *emmeans* and represents the estimated incidence rate for each group after accounting for the influence of covariates. For each symptom, Incidence Rate Ratios (IRRs) were calculated by dividing the IR in the probiotic group by that in the placebo group. An IRR < 1 indicates a lower incidence in the probiotic group compared to the placebo group, while an IRR > 1 indicates a higher incidence.

Values of *p* were corrected for multiple comparisons with a Tukey’s adjustment and considered statistically significant when ≤ 0.05.

### Fecal sample collection

The participants provided fecal samples at baseline and endpoint using the Fe-Col® Fecal Sample Collection kits (Alpha Laboratories, Hampshire, UK). Samples were stored refrigerated (for a maximum of 48 h) prior to transfer to the study center where samples were stored at − 80°C until analysis.

### Fecal DNA extraction and shotgun metagenomic sequencing

Genomic DNA was extracted from fecal samples using the Col® PowerFaecal Pro DNA Kit (Qiagen, Germany). The eluted genomic DNA was quantified using a Col® Fluorometer (Thermo Fisher Scientific, United States) and stored at − 20°C until analysis.

Shotgun metagenomic sequencing of the genomic DNA extracts was performed using the Illumina NovaSeq X platform (Novogene, China) with 150-nucleotide long paired-end reads. Raw sequence quality was assessed using FastQC (v0.11.6)^21^ before using Trimmomatic (v0.39)^22^ to remove adapter sequences, bases with low-quality scores (Phred score < 30) and reads shorter than 50 base pairs. Remaining reads were aligned against the GRCh38 human genome using Bowtie2 (v2.5.4)^23^ and any reads which mapped successfully were removed. Host-free reads were extracted using Samtools (v1.21)^24^ and BEDTools (v2.31.1).^25^

### Fecal microbiota analysis

#### Microbial abundance profiling

Taxonomic profiles of bacteria, archaea, fungi, and viruses were generated using Kraken2 (v.2.1.3)^26^ employing k-mer-based algorithms. Reads were classified against a Kraken2 custom database built from bacterial, archaeal, fungal, and viral genomes available in the NCBI RefSeq database (December 2024). Bracken (v3.1)^27^ was used to refine species-level abundance estimates. Downstream analyses were restricted to bacterial species with a relative abundance ≥ 0.1% in at least 10% of samples within each group and time-point.

#### Bacterial diversity

Bacterial species abundance tables were split by time-point to compare microbiota composition between probiotic and placebo groups at baseline and endpoint. Alpha diversity was calculated using the *phyloseq* R package v 1.50.0^28^ and compared using mixed-effects models with the same covariates as the questionnaire analysis (see Statistical analysis section). Bray–Curtis distance matrices were also generated with *phyloseq* and ordinated using Non-Metric Multidimensional Scaling (NMDS). Differences in community dispersion (mean distance of samples to the group centroid) were assessed using the *betadisper* and *permutest* functions in the *vegan* R package (v2.6–10).^29^ Community composition, reflected by the positioning of samples in ordination space, was compared using Permutational Multivariate Analysis of Variance (PERMANOVA) with 1000 permutations, adjusting for covariates in a multi-factor model.

#### Differential abundance

Differential abundance analysis was conducted using MaAsLin3 v 0.99.7^30^ on log-transformed relative abundance data without additional normalization, controlling for the same covariates. The Benjamini–Hochberg method was used to correct *p* values for multiple testing.

#### Detection of probiotic strains

The detection of the supplemented probiotic strains was performed using inStrain v1.9.1^31^ with short-read-derived whole genome assemblies serving as a reference.^32^ Detection required ≥ 99% of the reference genome to be covered by mapped reads and the population Average Nucleotide Identity (popANI) to exceed 99.999%. Correlative analyses were performed using pairwise Spearman’s rank coefficient analysis (GraphPad Prism, v10.20.2) where a Spearman’s correlation coefficient (r_s_) value of ≤ 0.29 indicates a weak correlation, 0.3 to 0.39 moderate correlation, 0.4 to 0.69 strong correlation and ≥ 0.7 very strong correlation. Values of *p* < 0.05 were considered significant.

## Results

### Recruitment and baseline characteristics

**Figure 2.**
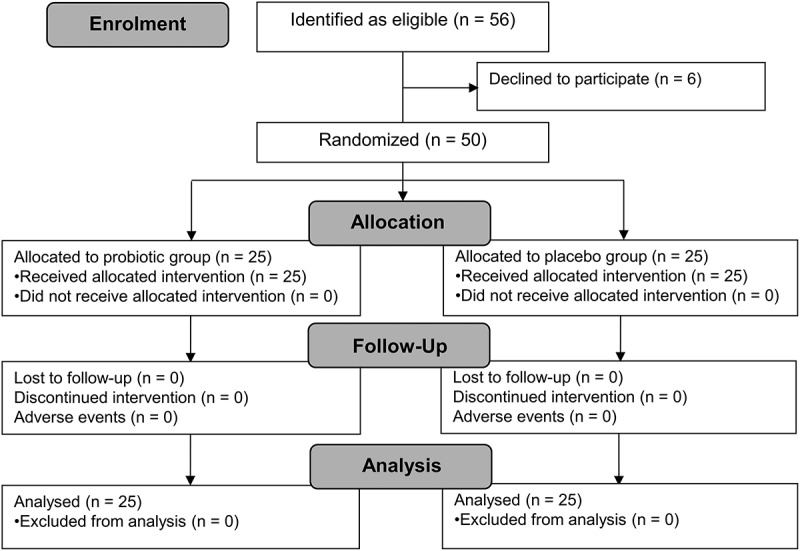
Study flow diagram.

A study flow diagram is provided in Figure 2. Of the fifty-six women contacted, 6 declined to participate and 50 were enrolled between 30/07/2024 and 05/08/2024. There were no drop-outs nor reported serious adverse effects. Compliance to the intervention exceeded 99% in both arms of the study.

The baseline characteristics of the study participants are presented in Table 1. During the study, none of the participants used contraceptive medicines and one probiotic group participant used an oral antibiotic. Two participants in the probiotic group used a non-hormonal vaginal cream/pessary. Fourteen women reported incidences of menstruation (bleeding) during the intervention period; 10 in the probiotic group and 4 in the placebo group.

**Table 1.** Baseline characteristics.

	Probiotic (n = 25)		Placebo (n = 25)	
	Mean	SD	Mean	SD
**Demographics & Anthropometry**				
Age, years	53.12	5.75	55.36	5.38
Height, meters	1.64	0.05	1.64	0.06
Body mass index, kg/m^2^	25.34	3.62	25.73	3.85
Systolic Blood Pressure, mmHg	121.40	7.31	125.80	8.39
Diastolic Blood Pressure, mmHg	78.72	5.22	79.88	4.39

Abbreviations: SD, standard deviation.

### Impact of daily supplementation on anxiety, depression, quality of life, sleep quality and overall well-being

Scores for the HADS (Anxiety and Depression), MRS, and AIS questionnaires at baseline, midpoint and endpoint are provided in Table 2. Baseline scores were similar between groups. At the midpoint and endpoint, the HADS Anxiety scores were significantly lower in the probiotic group compared to the placebo (−2.00, −35.9%; *p* = 0.0042 and −1.98, −39.7%; *p* = 0.0026, respectively) as were the MRS scores (−1.42, −15.7%; *p =* 0.0548 and −1.49, −18.5%; *p =* 0.0327, respectively). AIS scores were 10.7% and 17.5% lower in the probiotic group compared to the placebo at midpoint and endpoint, respectively, although the differences between groups did not reach statistical significance.

**Table 2.** Changes in HADS, MRS, AIS and OWBS over the duration of the study.

	Time	Adjusted Mean (unscaled) with 95% CI		Between Group Difference (95% CI)	Between Group Difference (%)	*p* value
		Probiotic (n = 25)	Placebo (n = 25)			
HADS Anxiety Score out of 21	BL	5.09 (4.14, 6.14)	5.11 (4.16, 6.16)	−0.02 (−1.46, 1.42)	−1.0	0.9769
	MP	3.57 (2.78, 4.46)	5.57 (4.58, 6.67)	−2.00 (−3.36, −0.64)	−35.9	0.0042
	EP	3.01 (2.29, 3.83)	4.99 (4.05, 6.03)	−1.98 (−3.25, −0.7)	−39.7	0.0026
HADS Depression Score out of 21	BL	4.48 (3.76, 5.2)	4.80 (4.08, 5.52)	−0.31 (−1.36, 0.73)	−6.7	0.5532
	MP	3.96 (3.24, 4.68)	4.04 (3.32, 4.76)	−0.07 (−1.12, 0.97)	−2.0	0.8879
	EP	3.96 (3.24, 4.68)	4.00 (3.28, 4.72)	−0.03 (−1.08, 1.01)	−1.0	0.9477
MRS (Quality of life) Score out of 44	BL	9.38 (8.31, 10.58)	9.83 (8.78, 11.01)	−0.45 (−1.99, 1.08)	−4.6	0.5609
	MP	7.60 (6.69, 8.62)	9.02 (7.96, 10.21)	−1.42 (−2.87, 0.03)	−15.7	0.0548
	EP	6.58 (5.76, 7.53)	8.07 (7.07, 9.21)	−1.49 (−2.85, −0.12)	−18.5	0.0327
AIS (Sleep Quality) Score out of 24	BL	5.13 (4.33, 5.93)	5.07 (4.27, 5.87)	0.07 (−1.08, 1.21)	1.2	0.9093
	MP	4.85 (4.05, 5.65)	5.43 (4.63, 6.23)	−0.57 (−1.72, 0.57)	−10.7	0.3240
	EP	4.05 (3.25, 4.85)	4.91 (4.11, 5.71)	−0.85 (−2.00, 0.29)	−17.5	0.1431
OWBS Score out of 100	BL	31.04 (28.07, 34.01)	31.65 (28.62, 34.68)	−0.61 (−4.8, 3.58)	1.9	0.7738
	MP	27.4 (24.43, 30.36)	32.53 (29.52, 35.54)	−5.13 (−9.33, −0.94)	−15.7	0.0168
	EP	25.59 (22.61, 28.57)	30.85 (27.84, 33.86)	−5.26 (−9.45, −1.07)	−17.1	0.0142

Abbreviations: BL, baseline; MP, midpoint; EP, endpoint; OR, odds ratio; CI, confidence interval; n, number of participants; HADS, Hospital Anxiety and Depression Scale; MRS, Menopause Rating Scale; AIS, Athens Insomnia Scale; OWBS, Overall Well-Being Score.

Overall Well-Being Score (OWBS) results are presented in Table 2 with a lower score representing better overall well-being. At baseline, OWBS in the groups were similar (*p =* 0.7739, Table 2). By the midpoint, there was a 15.7% difference between the groups favoring the probiotic group (probiotic: 27.4 [95% CI: 24.4, 30.4], placebo: 32.5 [95% CI: 29.5, 35.5]), indicating an improvement in overall well-being in the probiotic group (*p =* 0.0168) and this differential had further improved by the endpoint with a difference of 17.1% (probiotic: 25.6 [95% CI: 22.6, 28.6] vs placebo: 30.9 [95% CI: 27.8, 33.9], *p =* 0.0142).

Table 3 shows the odds ratios at endpoint, indicating the likelihood of participants in the probiotic group reporting less severe outcomes compared to those in the placebo group for each item of the MRS and AIS questionnaires. From the MRS questionnaire there were significantly better outcomes in the probiotic group for “*hot flushes”* (*p =* 0.0225), *“heart discomfort”* (*p =* 0.0322) and “*anxiety”* (*p =* 0.0381) together with improvements for *“irritability”* (*p =* 0.0674) and “*Dryness of vagina”* (*p =* 0.0911). For AIS, “*Sleepiness during the day”* (*p =* 0.0075) and *“Functioning during the day”* (*p =* 0.0422) were better for the probiotic group. There were no differences between the groups at baseline (Supplementary Table S1).

**Table 3.** Odds ratios (probiotic vs. placebo) at endpoint for the items of the MRS and AIS questionnaires.

Questionnaire/item	OR	Lower CI	Upper CI	*p* value
***MRS***				
Hot flushes, sweating:	7.69	1.33	43.48	0.0225
Heart discomfort:	5.81	1.16	29.41	0.0322
Sleep problems:	0.82	0.19	3.51	0.7910
Depressive mood:	1.68	0.28	9.90	0.5664
Irritability:	3.47	0.91	13.16	0.0674
Anxiety:	6.71	1.11	40.00	0.0381
Physical and mental exhaustion:	1.72	0.41	7.25	0.4563
Sexual problems:	2.11	0.47	9.43	0.3282
Bladder problems:	2.00	0.29	13.86	0.4836
Dryness of vagina:	6.85	0.73	62.50	0.0911
Joint and muscular discomfort:	0.76	0.17	3.50	0.7264
***AIS***				
Sleep induction:	1.59	0.35	7.25	0.5505
Awakening during the night:	0.60	0.14	2.59	0.4944
Final awakening earlier than desired:	3.17	0.70	14.29	0.1336
Total sleep duration:	0.65	0.16	2.66	0.5484
Overall quality of sleep (no matter how long you slept):	0.86	0.13	5.59	0.8725
Sense of well-being during day:	3.95	0.70	22.22	0.1184
Functioning (physical and mental) during the day:	6.90	1.07	45.45	0.0422
Sleepiness during the day:	12.66	1.97	83.33	0.0075

Abbreviations: OR, odds ratio; CI, confidence interval; MRS, Menopause Rating Scale; AIS, Athens Insomnia Scale.

### Digestive discomfort and other common issues

**Figure 3.**
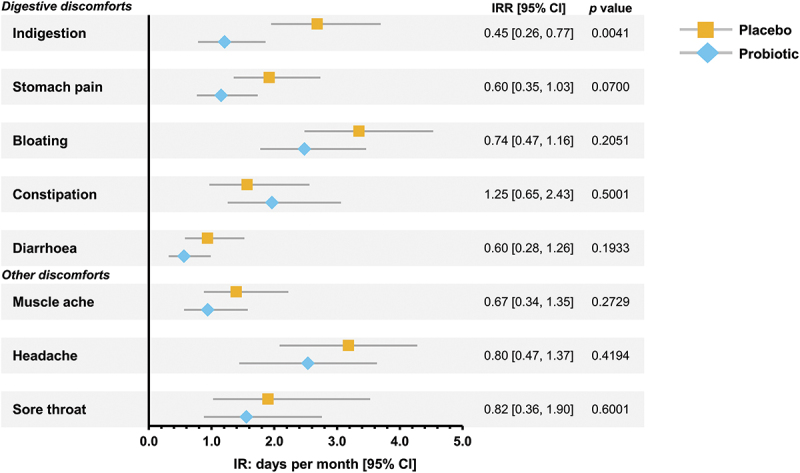
Incidence rates and rate ratios of digestive and other common discomforts. Data in forest plot is presented as adjusted mean days per month with 95% confidence intervals for 25 participants per group. Abbreviations: IR, incidence rate; IRR, incidence rate ratio; CI, confidence interval.

Incidence rates of digestive and other discomforts occurring over the duration of the intervention period are shown in Figure 3. Those in the probiotic group reported 55% less days with indigestion than in the placebo (*p =* 0.0041) and 40% less days with stomach pain (*p =* 0.0700). There were no significant between group differences in the rates of bloating, constipation, diarrhea, muscle ache, headache or sore throat between the probiotic and the placebo groups.

### Analysis of faecal microbiota

#### Bacterial diversity

**Figure 4.**
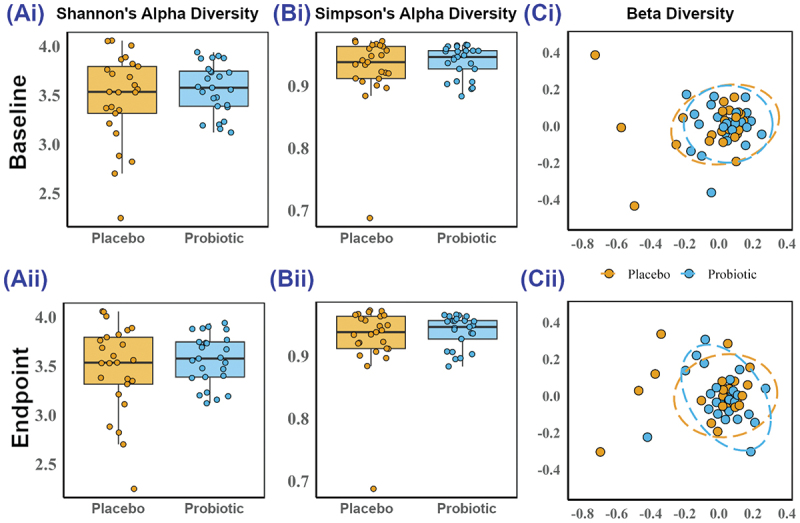
Diversity of the gut microbiota bacterial diversity. (A) Shannon’s alpha diversity index, (B) Simpson’s alpha diversity index and (C) beta diversity non-metric multidimensional scaling (NMDS) plots comparing the probiotic group with the placebo group at (*i*) baseline and (*ii*) endpoint. Each dot represents an individual participant (n = 25 per group). For (C), ellipses represent 95% confidence intervals around group centroids.

There were no between or within group differences at baseline or endpoint in either Shannon’s or Simpson’s alpha diversity (Figures 4A/B) or beta diversity (Figure 4C).

#### Differential abundance and supplemented strain detection

**Figure 5.**
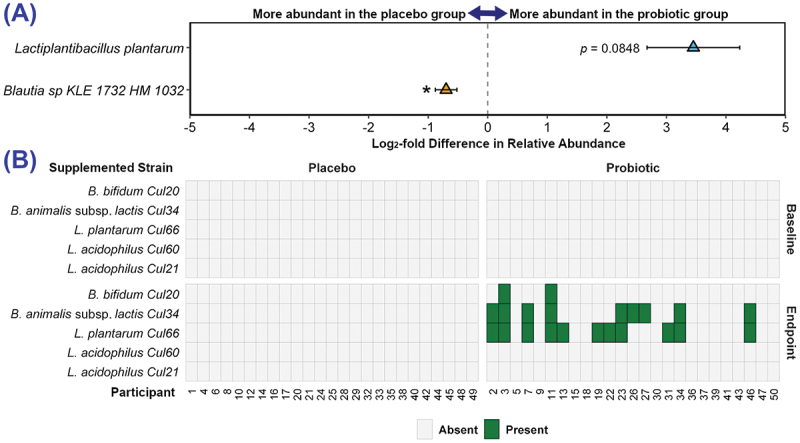
Changes in bacterial relative abundance and detection of supplemented strains. (A) Differentially abundant bacterial species between placebo and probiotic groups at endpoint. Data is presented as log₂-Fold difference in relative abundance with 95% confidence intervals. (B) Detection of the supplemented probiotic strains in fecal samples at baseline and endpoint. Values of *p* are: *p ≤0.05 or as stated.

The abundance of each bacterial species at baseline and endpoint was compared between groups. Figure 5A presents the species which were differentially abundant at endpoint; *Lactiplantibacillus plantarum* trended toward a higher abundance in the probiotic group (*p* = 0.0848) whereas, *Blautia* sp. KLE 1732 HM 1032 was more abundant in the placebo group (*p* = 0.0456). There were no significant differences between the groups at baseline.

All fecal samples were screened for the supplemented strains (Figure 5B) and none of the probiotic strains were detected in the placebo group but they were present in the probiotic group at endpoint. In 13 out of 25 of the probiotic group participants, at least one strain was detected; *Lactiplantibacillus plantarum* CUL66 was detected in 11 out of 25, *Bifidobacterium animalis* subsp. *lactis* CUL34 in 9, *Bifidobacterium bifidum* CUL20 in 2. Neither of the *Lactobacillus acidophilus* strains (CUL21/CUL60) was detected.

The detection of *L. plantarum* CUL66 in the probiotic group at endpoint was strongly and positively correlated with the abundance of the *L. plantarum* species (r_s_ = 0.525, *p* = 0.0070) and at the end point there was increased abundance of *L. plantarum* (Figure 5A).

The correlation of the total scores for the HADS Anxiety, HADS Depression, MRS and AIS questionnaires and OWBS for those participants in the probiotic group with at least one probiotic strain highlighted a moderate inverse correlation for HADS Depression (r_s_ = −0.31, *p* = 0.1299) and weak inverse correlations for MRS (r_s_ = −0.14, *p* = 0.8944) and OWBS (r_s_ = −0.12, *p* = 0.5610). Correlations for HADS Anxiety (r_s_ = 0.03, *p* = 0.03) and AIS (r_s_ = −0.01, *p* = 0.9788) were negligible.

Total scores for HADS Anxiety, HADS Depression, MRS, AIS and OWBS were also correlated with the detection of the individual probiotic strains and there were no significant associations (Supplementary Table S2).

## Discussion

Daily supplementation with the Lab4P probiotic resulted in a 40% reduction in anxiety, and a 20% improvement in quality of life which included reduced hot flushes, indications of improved sleep quality and reduced incidence of indigestion in women aged 45 to 65. At the end of the study in the probiotic group, *Blautia* sp. KLE 1732 was less abundant than in the placebo group and the Lab4P strains were detected in approximately half of the participants.

This population of healthy women did not present with any clinical symptoms of anxiety (HADS anxiety score < 8)^14^ and yet day-to-day anxiety levels were 40% lower in the probiotic group compared to the placebo by the end of the study. Merkouris *et al* concluded that probiotic supplementation can alleviate anxiety and depression, particularly for individuals presenting with milder symptoms.^33^ In previous studies with the Lab4P probiotic, we observed enhanced mood scores^11^ and reductions in anxiety scores.^12^

As a means of assessing quality of life we used the Menopause Rating Scale (MRS) which is a female specific questionnaire assessing a broad range of urogenital, somatic and psychological symptoms, ranging from hot flushes to sexual problems. The baseline MRS scores of < 12 for this cohort indicated a low symptom burden,^34^ but in the probiotic group there was a significant 20% reduction in score and this was associated with reduced hot flushes, heart discomfort, and levels of anxiety. Probiotic mediated reductions in MRS scores, hot flushes and heart palpitations have been observed elsewhere.^8,9^

Sleep quality tends to be disturbed as a consequence of aging and is widely recognized as contributory to decreased quality of life,^35^ impaired cognitive function and psychological distress.^36^ Sleep quality was measured using the Athens Insomnia Scale (AIS) and whilst the probiotic was not associated with significant improvements in overall score, there were item-level improvements in “Sleepiness during the day” and “Functioning (physical and mental) during the day.” Improved sleep quality with probiotic intake, including a reduction in “sleepiness on rising,” has been reported^37^ but results relating to sleep quality are inconsistent.^38,39^

The inclusion of HADS Depression, HADS Anxiety, MRS, and AIS questionnaires within the same study framework enabled us to generate an *Overall Well-Being Score*, integrating diverse psychological and physiological outcomes into a single metric. Notably, this analysis revealed a statistically significant 18% improvement in the probiotic group compared to placebo. Given the relative scarcity of research assessing probiotic benefits in mid-life women, the incorporation of similar analytical approaches in future trials (or retrospective analyses of existing data where applicable), maybe useful to better understand the broader well-being potential of probiotic supplementation in this underserved demographic.

Indigestion and stomach pains tend to become more of a problem with age^40^ and in the probiotic group there was a 55% reduction in the incidence rate of indigestion and a 40% reduction in the incidence of stomach pains. These findings support our previous work where there were reductions in indigestion and stomach pains^13^ and in Irritable Bowel Syndrome sufferers with high rates of gut problems we showed that the severity and incidence of stomach pains was reduced by probiotic supplementation.^12^

Analysis of the fecal microbiota revealed no probiotic-associated changes in diversity (richness and evenness) or community composition but at the endpoint, the probiotic group had a significantly lower abundance of *Blautia* sp. KLE 1732 HM 1032 and a higher abundance of *L. plantarum*. Increased levels of *Blautia* have been linked to the incidence of hot flushes and sleep disturbances,^41,42^ and whilst little is known about the mechanisms at play, lower levels observed in response to Lab4P may be related to the reduced symptomology. *L. plantarum* has been linked with lower levels of anxiety and stress and improvements in overall psychological well-being^43,44^ possibly via the ability to modulate levels of neuroactive molecules such as gamma-aminobutyric acid (GABA),^45^ brain-derived neurotrophic factor (BDNF)^46^ and cortisol.^47^

In this study we attempted for the first time to detect the presence of the Lab4P probiotic strains in the fecal samples. Using the techniques currently available to us we were able to detect at least one of the probiotic organisms (not *L. acidophilus*) in approximately half of the participants receiving the probiotic but correlative analysis did not find any significant associations between strain presence and the outcomes of HADS, MRS, AIS or OWBS in the participants receiving the probiotic. Theoretically, all strains should be detectable in all participants receiving the study products suggesting that there are limitations to the current method. The complete absence of the *L. acidophilus* strains clearly highlights the difficulty in drawing any conclusions from the data at this stage.

The study has strengths and limitations. Strengths include: *i*) the breadth of participant-completed questionnaires providing a holistic insight into participant well-being, and *ii*) the use of a free-living cohort, enhancing the translatability of the findings to general populations. Limitations of the study include: *i*) the absence of study arms receiving the probiotic alone and/or vitamins C and D and zinc alone and *ii*) the lack of data sourced on the menopausal status of the participants, that should be considered in future studies.

In summary, daily supplementation with the Lab4P probiotic over four months in free-living, middle-aged and healthy women (as evidenced by their low symptom burden) led to significant improvements in overall well-being. There were significant reductions in anxiety, hot flushes, tiredness, and gastrointestinal discomfort suggesting a potential role of probiotics to support women during the menopause transition (MT) period. The well-being improvements in the probiotic group were not associated with major shifts in microbiota composition but there was reduced *Blautia* abundance.

The outcomes from this exploratory study provide guidance for studies targeting women with more severe symptoms or those at specific stages of MT.

## Supplementary Material

ProWOME_Supplement2.docx

## Data Availability

Data is available from the corresponding author upon reasonable request. Sequence data generated during the study is deposited in the European Molecular Biology Laboratory (EMBL) nucleotide sequence database (https://www.ebi.ac.uk/ena.) under accession number: PRJEB89025

## Generative artificial intelligence (AI)

ChatGPT model 4o was used for coding assistance in R and language improvement. Generative AI was not used for idea generation or exploration.

## Health and safety

All mandatory laboratory health and safety procedures have been complied with in the course of conducting any experimental work reported in this study.

## References

[1] Fan Y, Pedersen O. Gut microbiota in human metabolic health and disease. Nat Rev Microbiol. 2021;19(1):55–13. doi: 10.1038/s41579-020-0433-9. 32887946

[2] Hasan N, Yang H. Factors affecting the composition of the gut microbiota, and its modulation. PeerJ. 2019;7:e7502. doi: 10.7717/peerj.7502. 31440436 PMC6699480

[3] Peters BA, Santoro N, Kaplan RC, Qi Q. Spotlight on the gut microbiome in menopause: current insights. Int J Womens Health. 2022;14:1059–1072. doi: 10.2147/IJWH.S340491. 35983178 PMC9379122

[4] Liaquat M, Minihane AM, Vauzour D, Pontifex MG. The gut microbiota in menopause: is there a role for prebiotic and probiotic solutions? Post Reprod Health. 2025;31(2):105–114. doi: 10.1177/20533691251340491. 40335047 PMC12209548

[5] Hill C, Guarner F, Reid G, Gibson GR, Merenstein DJ, Pot B, Morelli L, Canani RB, Flint HJ, Salminen S, Calder PC, Sanders ME. Expert consensus document. The international scientific association for probiotics and prebiotics consensus statement on the scope and appropriate use of the term probiotic. Nat Rev Gastroenterol Hepatol. 2014;11(8):506–514. doi: 10.1038/nrgastro.2014.66. 24912386

[6] Honda S, Tominaga Y, Espadaler-Mazo J, Huedo P, Aguiló M, Perez M, Ueda T, Sawashita J. Supplementation with a probiotic formula having β-glucuronidase activity modulates serum estrogen levels in healthy peri- and postmenopausal women. J Med Food. 2024;27(8):720–727. doi: 10.1089/jmf.2023.k.0320. 38742994

[7] Szulińska M, Łoniewski I, Skrypnik K, Sobieska M, Korybalska K, Suliburska J, Bogdański P. Multispecies probiotic supplementation favorably affects vascular function and reduces arterial stiffness in obese postmenopausal women-A 12-week placebo-controlled and randomized clinical study. Nutrients. 2018;10(11):10. doi: 10.3390/nu10111672. PMC6265939 30400570

[8] Ayubi E, Abdoli S, Mehrpooya M, Karami Z, Jenabi E, Ghaleiha A, Soltani F, Salehi AM. The effect of probiotic administration on the severity of menopausal symptoms and mental health of postmenopausal women: a triple-blind randomized controlled trial in the West of Iran. Menopause. 2025;32(2):166–173. doi: 10.1097/GME.0000000000002462. 39774869

[9] Lim EY, Lee SY, Shin HS, Lee J, Nam YD, Lee DO, Lee JY, Yeon SH, Son RH, Park CL, Heo YH, Kim YT. The effect of lactobacillus acidophilus YT1 (MENOLACTO) on improving menopausal symptoms: a randomized, double-blinded, placebo-controlled clinical trial. J Clin Med. 2020;9(7):2173. doi: 10.3390/jcm9072173. 32660010 PMC7408745

[10] Sawada D, Sugawara T, Hirota T, Nakamura Y. Effects of lactobacillus gasseri CP2305 on mild menopausal symptoms in middle-aged women. Nutrients. 2022;14(9):14. doi: 10.3390/nu14091695. PMC9101532 35565662

[11] Michael DR, Jack AA, Masetti G, Davies TS, Loxley KE, Kerry-Smith J, Plummer JF, Marchesi JR, Mullish BH, McDonald JAK, Hughes TR, Wang D, Garaiova I, Paduchová Z, Muchová J, Good MA, Plummer SF. A randomised controlled study shows supplementation of overweight and obese adults with lactobacilli and bifidobacteria reduces bodyweight and improves well-being. Sci Rep. 2020;10(1):4183 doi: 10.1038/s41598-020-60991-7. 32144319 PMC7060206

[12] Mullish BH, Michael DR, Dabcheva M, Webberley TS, Coates N, John DA, Wang D, Luo Y, Plummer SF, Marchesi JR. A double-blind, randomized, placebo-controlled study assessing the impact of probiotic supplementation on the symptoms of irritable bowel syndrome in females. Neurogastroenterol Motil. 2024;36(4):e14751. doi: 10.1111/nmo.14751. 38287443

[13] Mullish BH, Michael DR, Webberley TS, John D, Ramanathan G, Plummer SF, Wang D, Marchesi JR. The gastrointestinal status of healthy adults: a *post hoc* assessment of the impact of three distinct probiotics. Benef Microbes. 2023;14(3):1–14. doi: 10.3920/BM2022.0092. 37026364

[14] Zigmond AS, Snaith RP. The hospital anxiety and depression scale. Acta Psychiatrica Scandinavica. 1983;67(6):361–370. doi: 10.1111/j.1600-0447.1983.tb09716.x. 6880820

[15] Schneider HP, Heinemann LA, Rosemeier HP, Potthoff P, Behre HM. The Menopause Rating Scale (MRS): reliability of scores of menopausal complaints. Climacteric. 2000;3(1):59–64. doi: 10.3109/13697130009167600. 11910611

[16] Soldatos CR, Dikeos DG, Paparrigopoulos TJ. Athens Insomnia Scale: validation of an instrument based on ICD-10 criteria. J Psychosom Res. 2000;48(6):555–560. doi: 10.1016/S0022-3999(00)00095-7. 11033374

[17] Brooks M, Kristensen K, van Benthem K, Magnusson A, Berg C, Nielsen A, Skaug H, Mächler M, Bolker B. glmmTMB balances speed and flexibility among packages for zero-inflated generalized linear mixed modeling. The R Journal. 2017;9(2):378–400. doi: 10.32614/RJ-2017-066.

[18] Hartig F. Dharma: residual diagnostics for hierarchical (multi-level/mixed) regression models. R package; 2024.

[19] Lenth R. Emmeans: estimated marginal means, aka least-squares means. R package; 2025.

[20] Christensen R. Ordinal: regression models for ordinal data. R package; 2023.

[21] Andrews S. FastQC: a quality control tool for high throughput sequence data. 2010.

[22] Bolger AM, Lohse M, Usadel B. Trimmomatic: a flexible trimmer for illumina sequence data. Bioinformatics. 2014;30(15):2114–2120. doi: 10.1093/bioinformatics/btu170. 24695404 PMC4103590

[23] Langmead B, Salzberg SL. Fast gapped-read alignment with Bowtie 2. Nat Methods. 2012;9(4):357–359. doi: 10.1038/nmeth.1923. 22388286 PMC3322381

[24] Li H, Handsaker B, Wysoker A, Fennell T, Ruan J, Homer N, Marth G, Abecasis G, Durbin R. The sequence alignment/map format and SAMtools. Bioinformatics. 2009;25(16):2078–2079. doi: 10.1093/bioinformatics/btp352. 19505943 PMC2723002

[25] Quinlan AR, Hall IM. Bedtools: a flexible suite of utilities for comparing genomic features. Bioinformatics. 2010;26(6):841–842. doi: 10.1093/bioinformatics/btq033. 20110278 PMC2832824

[26] Wood DE, Lu J, Langmead B. Improved metagenomic analysis with Kraken 2. Genome Biol. 2019;20(1):257. doi: 10.1186/s13059-019-1891-0. 31779668 PMC6883579

[27] Lu J, Breitwieser FP, Thielen P, Salzberg SL. Bracken: estimating species abundance in metagenomics data. Peer J Comput Sci. 2017;3:3. doi: 10.7717/peerj-cs.104. PMC12016282 40271438

[28] McMurdie PJ, Holmes S, Watson M. Phyloseq: an R package for reproducible interactive analysis and graphics of microbiome census data. PLOS ONE. 2013;8(4):e61217. doi: 10.1371/journal.pone.0061217. 23630581 PMC3632530

[29] Oksanen J, Simpson GL, Blanchet FG, Kindt R, Legendre P, Minchin PR, O’Hara RB, Solymos P, Stevens MHM, Szoecs E, Wagner H, Barbour M, Bedward M, Bolker B, Borcard D, Borman T, Carvalho G, Chirico M, De Caceres M, Durand S, Evangelistra HBA. Vegan: community ecology package. ; 2025.

[30] Nickols W, Nearing J. maaslin3: “refining and extending generalized multivariate linear models for meta-omic association discovery”. R package; 2025. 10.1038/s41592-025-02923-9 PMC12982127 41540124

[31] Olm MR, Crits-Christoph A, Bouma-Gregson K, Firek BA, Morowitz MJ, Banfield JF. inStrain profiles population microdiversity from metagenomic data and sensitively detects shared microbial strains. Nat Biotechnol. 2021;39(6):727–736. doi: 10.1038/s41587-020-00797-0. 33462508 PMC9223867

[32] Baker LM, Davies TS, Masetti G, Hughes TR, Marchesi JR, Jack AA, Joyce TSC, Allen MD, Plummer SF, Michael DR, Ramanathan G, Del Sol R, Facey PD. A genome guided evaluation of the Lab4 probiotic consortium. Genomics 113 4028–4038 doi:10.1016/j.ygeno.2021.08.007. 2021. 34391865

[33] Merkouris E, Mavroudi T, Miliotas D, Tsiptsios D, Serdari A, Christidi F, Doskas TK, Mueller C, Tsamakis K. Probiotics’ effects in the treatment of anxiety and depression: a comprehensive review of 2014-2023 clinical trials. Microorganisms. 2024;12(2):12. doi: 10.3390/microorganisms12020411. PMC10893170 38399815

[34] Masjoudi M, Amjadi MA, Leyli EKN. Severity and frequency of menopausal symptoms in middle aged women, Rasht, Iran. J Clin Diagnostic Res. 2017;11:Qc17–qc21. doi: 10.7860/JCDR/2017/26994.10515. PMC5620854 28969213

[35] Miner B, Kryger MH. Sleep in the aging population. Sleep Med Clinics. 2017;12(1):31–38. doi: 10.1016/j.jsmc.2016.10.008. PMC5300306 28159095

[36] Corbo I, Forte G, Favieri F, Casagrande M. Poor sleep quality in aging: the association with mental health. Int J Environ Res Pub Health Public Health. 2023;20(3):1661. doi: 10.3390/ijerph20031661. PMC9914898 36767029

[37] Ito H, Tomura Y, Kitagawa Y, Nakashima T, Kobanawa S, Uki K, Oshida J, Kodama T, Fukui S, Kobayashi D. Effects of probiotics on sleep parameters: a systematic review and meta-analysis. Clin Nutr ESPEN. 2024;63:623–630. doi: 10.1016/j.clnesp.2024.07.006. 39094854

[38] Gil-Hernández E, Ruiz-González C, Rodriguez-Arrastia M, Ropero-Padilla C, Rueda-Ruzafa L, Sánchez-Labraca N, Roman P. Effect of gut microbiota modulation on sleep: a systematic review and meta-analysis of clinical trials. Nutr Rev. 2023;81(12):1556–1570. doi: 10.1093/nutrit/nuad027. 37023468

[39] Santi D, Debbi V, Costantino F, Spaggiari G, Simoni M, Greco C, et al. Microbiota composition and probiotics supplementations on sleep quality—A systematic review and meta-analysis. Clocks Sleep. 2023;5:770–792. doi: 10.3390/clockssleep5040050. 38131749 PMC10742335

[40] Gallo A, Pellegrino S, Pero E, Agnitelli MC, Parlangeli C, Landi F, Montalto M. Main disorders of gastrointestinal tract in older people: an overview. Gastrointestinal Disord. 2024;6(1):313–336. doi: 10.3390/gidisord6010022.

[41] Xie X, Song J, Wu Y, Li M, Guo W, Li S, Li Y. Study on gut microbiota and metabolomics in postmenopausal women. BMC Women’s Health. 2024;24:608. 39548431 10.1186/s12905-024-03448-7 PMC11566192

[42] Haimov I, Magzal F, Tamir S, Lalzar M, Asraf K, Milman U, Agmon M, Shochat T. Variation in gut microbiota composition is associated with sleep quality and cognitive performance in older adults with Insomnia. Nat Sci Sleep. 2022;14:1753–1767. doi: 10.2147/NSS.S377114. 36225322 PMC9550024

[43] Önning G, Montelius C, Hillman M, Larsson N. Intake of *lactiplantibacillus plantarum* HEAL9 improves cognition in moderately stressed subjects: a randomized controlled study. Nutrients. 2023;15(15):15. doi: 10.3390/nu15153466. PMC10421450 37571403

[44] Chan HHY, Siu PLK, Choy CT, Chan UK, Zhou J, Wong CH, Lee YW, Chan HW, Tsui JCC, Loo SKF, Tsui SKW. Novel multi-strain E3 probiotic formulation improved mental health symptoms and sleep quality in Hong Kong Chinese. Nutrients. 2023;15(24):5037. doi: 10.3390/nu15245037. 38140296 PMC10745623

[45] Grant AD, Erfe MCB, Delebecque CJ, Keller D, Zimmerman NP, Kazaryan A, Oliver PL, Moos J, Luna V, Craft N. GABA probiotic Lactiplantibacillus plantarum Lp815 improves sleep, anxiety and increases urinary GABA: a randomized, double-blind, placebo-controlled study. medRxiv 2025:2025.04.14.25325830.

[46] Sun X, Zhang H-F, Ma C-L, Wei H, Li B-M, Luo J. Alleviation of anxiety/Depressive-like behaviors and improvement of cognitive functions by Lactobacillus plantarum WLPL04 in chronically stressed mice. Can J Infect Dis Med Microbiol. 2021;2021:1–11. doi: 10.1155/2021/6613903. PMC7868149 33603935

[47] Chong HX, Yusoff NAA, Hor YY, Lew LC, Jaafar MH, Choi SB, Yusoff MSB, Wahid N, Abdullah MFIL, Zakaria N, Ong LK, Park YH, Liong MT. *Lactobacillus plantarum* DR7 alleviates stress and anxiety in adults: a randomised, double-blind, placebo-controlled study. Benef Microbes. 2019;10(4):355–374. doi: 10.3920/BM2018.0135. 30882244

