# Supplementary material for: Probiotic supplementation improves well-being and anxiety in healthy women: An exploratory, randomized, double-blind, placebo-controlled study
Source: Gut Microbes Rep. 2025 Sep 5;2(1):2543125. doi: 10.1080/29933935.2025.2543125 (PMC12940106; doi:10.1080/29933935.2025.2543125)
Supplement: ProWOME_Supplement2.docx [file KGMR_A_2543125_SM1598.docx]

**SUPPLEMENTARY INFORMATION**

**Probiotic supplementation improves the well-being and anxiety in healthy women: An exploratory, randomised, double-blind, placebo-controlled study**

**Supplementary Figure S1: Weekly Well-being Diary**

**
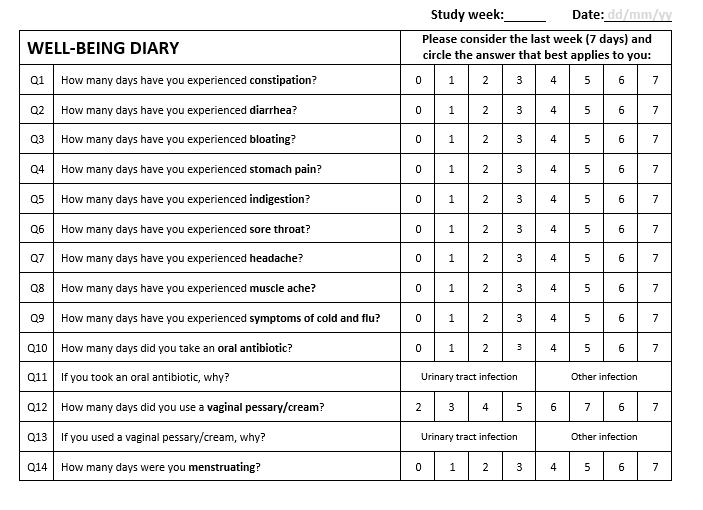
**

**Supplementary Table S1: Odds ratios (Probiotic vs. Placebo) at baseline for the items of the MRS and AIS questionnaires.**

| **Questionnaire/item** | | **OR** | **Lower CI** | **Upper CI** | ***p* value** |
| --- | --- | --- | --- | --- | --- |
| ***MRS*** | |  |  |  |  |
|  | Hot flushes, sweating: | 0.95 | 0.22 | 4.10 | 0.9459 |
|  | Heart discomfort: | 0.84 | 0.20 | 3.46 | 0.8103 |
|  | Sleep problems: | 0.63 | 0.16 | 2.49 | 0.5141 |
|  | Depressive mood: | 1.33 | 0.26 | 6.90 | 0.7334 |
|  | Irritability: | 0.92 | 0.27 | 3.18 | 0.5664 |
|  | Anxiety: | 0.90 | 0.18 | 4.42 | 0.9004 |
|  | Physical and mental exhaustion: | 0.69 | 0.16 | 3.03 | 0.6218 |
|  | Sexual problems: | 0.99 | 0.25 | 3.83 | 0.9870 |
|  | Bladder problems: | 0.97 | 0.15 | 6.29 | 0.9746 |
|  | Dryness of vagina: | 1.20 | 0.18 | 8.00 | 0.8510 |
|  | Joint and muscular discomfort: | 1.69 | 0.39 | 7.30 | 0.4789 |
| ***AIS*** | |  |  |  |  |
|  | Sleep induction: | 1.34 | 0.37 | 4.88 | 0.6623 |
|  | Awakening during the night: | 0.78 | 0.19 | 3.24 | 0.7298 |
|  | Final awakening earlier than desired: | 0.67 | 0.15 | 2.99 | 0.6018 |
|  | Total sleep duration: | 1.12 | 0.29 | 4.31 | 0.8694 |
|  | Overall quality of sleep (no matter how long you slept): | 0.64 | 0.10 | 4.22 | 0.6389 |
|  | Sense of well-being during day: | 1.04 | 0.22 | 4.90 | 0.9591 |
|  | Functioning (physical and mental) during the day: | 1.04 | 0.19 | 5.65 | 0.9675 |
|  | Sleepiness during the day: | 1.75 | 0.33 | 9.35 | 0.5108 |

**Supplementary Table S2. Association’s between the presence of the Lab4P probiotic strains and the outcomes of the well-being questionnaires within the probiotic group at endpoint**

|  | **Spearman's coefficient (r_s_)*** | ***p* value** |
| --- | --- | --- |
| **CUL 66** |  |  |
| HADS Anxiety | -0.08 | 0.7080 |
| HADS Depression | -0.22 | 0.2859 |
| MRS | -0.18 | 0.3907 |
| AIS | -0.01 | 0.9573 |
| OWBS | -0.13 | 0.5228 |
|  |  |  |
| **CUL 34** |  |  |
| HADS Anxiety | -0.02 | 0.9120 |
| HADS Depression | -0.34 | 0.1008 |
| MRS | -0.19 | 0.3745 |
| AIS | -0.13 | 0.5223 |
| OWBS | -0.17 | 0.4073 |
|  |  |  |
| **CUL 20** |  |  |
| HADS Anxiety | -0.34 | 0.0964 |
| HADS Depression | 0.04 | 0.8432 |
| MRS | 0.03 | 0.8839 |
| AIS | 0.29 | 0.1609 |
| OWBS | 0.04 | 0.8461 |

*Correlative analyses were performed using pairwise Spearman’s rank coefficient (GraphPad Prism, Version 10.20.2). Spearman’s r_s_ values of ≤0.29 indicate a weak correlation, 0.3 to 3.9 indicate a moderate correlation, 0.4 to 0.69 indicate a strong correlation and ≥ 0.7 indicate a very strong correlation. Values of *p*<0.05 were considered significant. *Abbreviations:* HADS, Hospital Anxiety and Depression Scale; MRS, Menopause Rating Scale; AIS, Athens Insomnia Scale; OWBS, Overall Well-Being Score.
